# The epidemiology of tuberculosis-associated hyperglycemia in individuals newly screened for type 2 diabetes mellitus: systematic review and meta-analysis

**DOI:** 10.1186/s12879-020-05512-7

**Published:** 2020-12-09

**Authors:** Sonia Menon, Rodolfo Rossi, Alfred Dusabimana, Natasha Zdraveska, Samit Bhattacharyya, Joel Francis

**Affiliations:** 1 Global Health Institute, University of Antwerp, Antwerp, Belgium; 2 Department of Public Health, Institute of Tropical Medicine, Sint Rochusstraat 43, 2000 Antwerp, Belgium; 3 International Committee of the Red Cross, Geneva, Switzerland; 4 Specialized hospital for Geriatric and Palliative medicine, Skopje, Republic of North Macedonia; 5 Department of Mathematics, School of Natural Sciences, Shiv Nadar University, Uttar Pradesh, India; 6 Department of Family Medicine and Primary Care, School of Clinical Medicine, Faculty of Health Sciences, University of the Witwatersrand, Johannesburg, South Africa

**Keywords:** Tuberculosis, Diabetes mellitus, TB-associated hyperglycemia, Screening

## Abstract

**Background:**

There is scarce evidence that tuberculosis (TB) can cause diabetes in those not previously known to be diabetic. Whilst the World Health Organization (WHO) recommends screening for Diabetes Mellitus (DM) at the onset of TB treatment, nevertheless, it remains to be elucidated which patients with TB-associated hyperglycemia are at higher risk for developing DM and stand to benefit from a more regular follow-up. This review aims to firstly quantify the reduction of newly detected hyperglycemia burden in TB patients who are on treatment over time; secondly, determine the burden of TB-associated hyperglycemia after follow-up, and thirdly, synthesize literature on risk factors for unresolved TB-associated hyperglycemia in previously undiagnosed individuals.

**Methods:**

We searched PUBMED, EMBASE, SCOPUS, and Global Health for articles on TB-associated hyperglycemia up to September 30th, 2019. Search terms included Tuberculosis and hyperglycemia/DM, and insulin resistance. We appraised studies, extracted data, and conducted a meta-analysis to assess the change of the burden of hyperglycemia in prospective studies. The review is registered in the PROSPERO database (CRD42019118173).

**Results:**

Eleven studies were included in the meta-analysis yielding a total of 677 (27,3%) of patients with newly detected hyperglycemia at baseline. The mean quality score of eligible studies using the Newcastle-Ottawa Quality Assessment Scale was 7.1 out of 9 (range 6-9). The pooled unresolved new cases of hyperglycemia at the end of follow up was 50% (95% CI: 36–64%) and the total pooled burden of hyperglycemia at 3–6 months of follow up was 11% (95% CI: 7–16%), with both estimates displaying a high heterogeneity, which remained significant after performing a sub-analysis by DM diagnostic method and 3 months of follow up. As only 2 studies explored risk factors for unresolved hyperglycemia, no meta-analysis was performed on risk factors.

**Conclusion:**

Our meta-analysis showed that although in half of the patients with newly observed hyperglycemia at baseline, it remained unresolved at a follow-up of 3 to 6 months, the total burden of hyperglycemia is slightly above 10%, 3 months after initiating TB treatment. Studies are warranted to assess whether risk factors including HIV positivity, smoking, and extensive pulmonary TB disease put patients at higher risk for DM.

**Supplementary information:**

**Supplementary information** accompanies this paper at 10.1186/s12879-020-05512-7.

## Background

Tuberculosis (TB), one of the most devastating communicable diseases in humans continues to take its toll across the globe. According to the World Health Organization (WHO), in 2018, TB was among the top 10 causes of death worldwide [1]. The WHO also estimated that about one quarter of the global population has latent TB, i.e. is infected by the TB causative agent, *Mycobacterium tuberculosis*, although the lifetime risk for this population to develop active and infectious TB is 5–15% [1], whilst HIV-infected people with malnutrition or diabetes, or tobacco users are at higher risk of developing active TB [1]. Active TB is generally classified as pulmonary or extra-pulmonary TB, with the former representing 85% of the total confirmed cases in 2017 [2]. Seven countries accounted for 64% of the total burden, with India facing the largest burden, followed by Indonesia, China, Philippines, Pakistan, Nigeria and South Africa [3].

First-line anti-TB drugs include, isoniazid, rifampicin, pyrazinamide, and ethambutol [4]. After 3 months of drug treatment for pulmonary TB caused by drug susceptible bacteria, 90–95% of patients will have negative sputum and show clinical improvement [4]. However, multidrug-resistant TB (MDR-TB) remains a grave problem. According to the WHO 2018 report, there were 484,000 new cases with resistance to rifampicin – the most effective first-line drug, of which 78% had MDR-TB [1].

At the same time, diabetes mellitus (DM) is steadily establishing global dominance as well. The WHO has reported an increase in the number of adults over 18 years of age with DM type 2 from 108 to 422 million in a span of 3 decades – (1980 to 2014) (4.7–8.5% prevalence) [5]. The majority of diabetes cases worldwide are type 2 DM. Unlike type 1 DM, which results from absolute insulin deficiency due to beta-cell loss, in individuals with type 2 DM the pancreas usually still produces some insulin. Excess body weight, obesity and physical inactivity have conspired to escalate rates of type 2 diabetes worldwide [6]. With the increase of the human life expectancy, more cases are likely to be diagnosed. DM carries with it an array of micro and macro vascular complications, which can lead to visual impairment, kidney failure, heart attacks, stroke and lower limb amputation [7].

Diabetics are 3 times more likely to succumb to infectious conditions including TB [8], in great part due to the weakened immune defenses as a result of the diabetes. Conversely, stress hyperglycemia associated with TB can ensue from an intricate interplay between disturbed cytokine and hormone production, resulting in excessive hepatic glucose production and insulin resistance [9], an interference which can last for 1 month or longer [10].

Due to the burgeoning of DM and TB cases in low- and middle-income countries, the WHO and the International Union Against Tuberculosis and Lung Disease (IUATLD) established a collaborative framework which promotes bidirectional screening including testing for TB among people with DM. Although the WHO recommends screening for DM at the onset of TB treatment, it is unknown which patients will be at risk for developing DM and would thus benefit from a more regular follow-up.

Our study aims to systematically review and synthesize the literature on the epidemiology of TB associated hyperglycemia with no date limit. Specifically, it aims to quantify the percentage of unresolved cases of hyperglycemia in TB patients receiving treatment over time. Secondly, it aims to determine the total burden of TB associated hyperglycemia at 3–6 months of follow-up. Thirdly, it aims to synthesize the literature on risk factors for the above progression. We therefore conducted a meta-analysis to quantify and numerically summarize the findings. Finally, both our narrative review and meta-analysis allowed us to identify clinical, epidemiological and public health research gaps. Our protocol has been registered with PROSPERO (CRD42019118173) https://www.crd.york.ac.uk/PROSPERO/display_record.php?RecordID=118173 and published in BMC Systematic reviews [11].

## Methods

### Literature search strategy

Our search strategy, selection of publications and the reporting of results for the review was conducted in accordance with the PRISMA guidelines [12]. The field of research terms included the thematic of TB and DM and their related field. We searched PUBMED, EMBASE, SCOPUS, and Global Health without any language and from 1960 onwards. Reference lists of all retrieved articles was checked to identify further eligible publications (Supplemental file: Search strategy).

### Eligibility criteria

Only peer reviewed publications were included. Eligible studies included those reporting on TB-associated hyperglycemia and following patients prospectively to explore the trajectory of hyperglycemia. Only prospective studies were eligible if they distinguished newly detected cases of hyperglycemia from known cases of DM at baseline and if follow up period was more 3 months or longer, case control studies were included provided it appeared clear that the exposure preceded the outcome. Case-series, case studies and cross-sectional studies were not eligible. For the third objective, we documented risk factors for unresolved hyperglycemia. Participants of interest were individuals with newly observed hyperglycemia following TB diagnosis/TB treatment registration.

Three co-authors gave independent opinion on whether or not to include a study; consensus was sought with all members of the entire research team. SM and AD resolved the discrepancies on eligible studies and extracted data through a discussion with RR and JF. In case of any missing information or need for clarification, respective study authors were contacted via email. We conducted this systematic review and meta-analysis based on a pre-defined search protocol that conformed to the criteria established by the Meta-Analysis of Observational Studies in Epidemiology (MOOSE) group and reported our findings in accordance with the PRISMA statement [12, 13] (Supplemental file: PRISMA statement).

### Exposure

Pulmonary TB disease defined by a positive chest X-ray and TB culture in patients of all ages.

### Outcome

TB-associated hyperglycemia was defined by presence of impaired fasting glycemia - IFG (fasting plasma glucose (FPG) levels ≥100 mg/dl (5.6 mmol/L) or impaired glucose tolerance - IGT (2-h oral glucose tolerance test (OGTT) of ≥140 mg/dl (7.8 mmol/L) [14] and hemoglobin A1C (HbA1c) ≥ 5.7% [15]. SM and AD independently reviewed and critically evaluated all identified studies for inclusion. Prospective studies with a follow up period of less than 3 months were not eligible as patients may still have positive sputum within this timeframe and we wanted to reduce interference from stress-related hyperglycemia [10]. Unresolved hyperglycemia was defined as lingering hyperglycemia from 3 months onwards. In all eligible studies, the furthest endpoint was considered.

### Data extraction and management

SM and AD recorded the following items: first author, study period, publication year, country, study design, study population, total sample size, mean and median age, method of diagnosis of TB, time of follow-up, number and percentage of newly detected hyperglycemia cases at baseline and at follow-up, glucose impairment status when diagnosed with TB, method used to ascertain persistent hyperglycemia, risk factors for persistent hyperglycemia using the reported ratios of measures of association with 95% confidence intervals (95% CI). For old irretrievable studies prior to 1980, medical librarians were consulted to inquire whether these could be accessed.

### Assessment of quality of the studies

SM and AD assessed the quality and risk of bias for each study included in the qualitative analysis. The Newcastle-Ottawa Quality Assessment Scale (NOS) was adapted to the research objectives of this systematic review for the evaluation of quality. Each study was assessed for good standards based on selection (maximum 4 points), comparability (maximum two points) and exposure (maximum 3 points). As we were interested in the follow-up of newly detected hyperglycemia cases, we allocated a point for a loss to follow up of 10% or lower [16] and no points when it was unclear if there was loss to follow up or whether it referred to this group. If the multivariate analysis performed within a study was not relevant to our research question, a point was not awarded for the item “study controls for at least 3 additional risk factors”. At the end, the mean score was calculated.

### Statistical analysis

The primary endpoint was the percentage of unresolved burden of TB associated hyperglycemia after follow-up, which was defined as per the criteria given above. This was calculated as the hyperglycemic cases at follow-up divided by the initial number of hyperglycemic cases, which was then transformed into a percentage. The secondary endpoint was the burden of TB-associated hyperglycemia at follow-up, which was calculated by dividing the cases of hyperglycemia 3–6 months by the size of study population at baseline. The cases of loss to follow up were subtracted from the size of study population at baseline.

We used the Der Simonian and Laird random-effects model to pool the effect size. The I^2^ test, which quantifies the proportion of total variability in effect size due to between study variation, was used to assess heterogeneity. Values of 50–75% represented substantial heterogeneity and over 75% represent, considerable heterogeneity. Potential causes of heterogeneity were explored by performing sensitivity analyses, excluding studies with high overall risk of bias. With regards to the three different diagnostic tests described above and the different periods of follow up, a sub analysis was performed by diagnostic test and by a follow- up period of 3 months compared to a followed up period of over three to 6 months.

To assess the robustness of pooled reduction of hyperglycemia, we performed a leave-one-out sensitivity analysis by iteratively removing one study at a time while recalculating the percentage. Forest plots were produced to show the pooled estimates. Analysis was undertaken using STATA version 16 (Corporation, College Station, TX, USA). The STATA command *Metaprop* was used to calculate the effect size.

## Results

### Search results

**Fig. 1:**
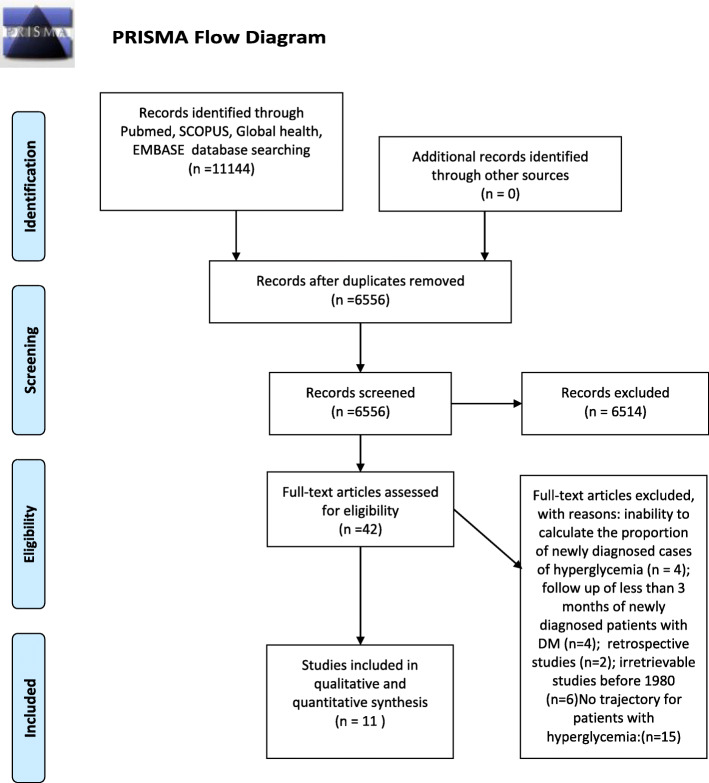
Prisma Flow Diagram

On December 19th, 2019, we retrieved 11,144 studies from PUBMED, EMBASE, SCOPUS, and Global Health of which 4588 were duplicates. Forty-two studies were eligible for full text screening. Finally, 31 studies were excluded after full text screening due to inability to calculate the proportion of newly detected cases of hyperglycemia (*n* = 4), follow up of less than 3 months of newly detected hyperglycemic patients (*n* = 4), retrospective study (*n* = 2), and no trajectory described for newly detected patients with hyperglycemia (*n* = 15), irretrievable studies before 1990 (*n* = 6) (Fig. 1: Flow diagram). Finally, 11 studies were eligible for inclusion.

### Study and participant characteristics

Study and participant characteristics of the prospective population-based cohort studies are summarized in Table 1. Six studies were conducted in Asia [17–22], 4 in sub-Saharan Africa [23–26] and 1 in Middle East [27] (Table 1). Sample size of newly detected hyperglycemic patients ranged from 9 to 296 and the mean age of study participants ranged from 29.4 to 43 years. In total, there were 437 HIV-infected patients. In one study, the patients with newly detected hyperglycemia were treated if their hyperglycemia persisted after 3 months [21], therefore a follow- up period of 3 months was used to measure end line hyperglycemia instead of 6 months.

**Table 1:** Description of the studies included in the systematic review and meta- analysis

Author	Diagnostic	Sample size	Age	Mean/median baseline blood glucose of newly screened patients for type 2 DM	n: newly detected hyperglycemia at baseline	n: unresolved hyperglycemia and %	Duration of follow up	Independent risk factors	Loss to follow up
Singh et al. (India, 1984) [17]	OGTT	52	Range 19–60 years with a mean age of 30.5 years	NA	23	6 (26%)	3 months		No loss to follow up
Kishore et al. (India,1971)	OGTT	90	Mean age of patients: 29.4	NA	19	5 (26%)	3 months		No loss to follow up
Olubuyo et al. (Nigeria, 1990) [18]	OGTT	54	Mean age of patients: 34.9 (SD: 14.4)	NA	23	7 (30%)	3 months		No loss to follow up
Kornfeld et al. (India, 2016) [19]	OGTT	153	Mean age of patients with newly diagnosed DM: 46.7 (± 10.7)	HbA1c: 6.8 (6.2–9.3)	33	27 (82%)	3 months		Loss to follow up (*n* = 24); unclear which patients
Basoglu et al. (Turkey, 1999) [20]	OGTT	58	Mean age of patients: 41.9 (15–82 years)	NA	11	10 (91%)	3 months		Loss to follow up (*n* = 17); unclear which patients
Boillat et al. (Tanzania, 2016)	AIC	530	Mean age of patients 35.9 (SD: 12)	NA	296	86 (29%)	5 months		Loss to follow up (*n* = 15); unclear which patients
Gupte et al. (India, 2018) [21]	AIC	392	Median age of patients: 31 (IQR: 23–44)	Median 8.5% (IQR: 6.7–11.5)	30	23 (77%)	6 months		No loss to follow up
Aftab et al. (Pakistan, 2017) [22]	FPG	462	Mean age of patients with newly diagnosed DM: 43 (95% CI: 41–45)	NA	195	74 (38%)	6 months		Unclear if there is loss to follow up
Lin et al. (China, 2017) [23]	FPG	270	Mean age of patients: 42.1 years	NA	13	9 (69%)	6 months	Association between HIV+, smoking and unstable blood glucose levels during TB treatment (aOR: 6.67, 95% CI: 1.24–35.71) and (aOR: 2.66, 95% CI: 1.37–5.2) respectively.	No loss to follow up
Kubjane et al. (South Africa, 2019)	AIC	390	Median age of patients with newly diagnosed DM: 36 (IQR: 30–43)	Median HbA1c: 6.7 (IQR: 6.5–7)	25	6 (24%)	3 months	HIV+ patients have a higher odds of being newly diagnosed with DM at baseline (OR:1.7; 95% CI: 1.0–2.9	Loss to follow up (*n* = 6)
Philips et al. (South Africa, 2017) [24]	FPG	29	Mean age of patients 33.95 (SD: 12.02)	Mean fasting insulin 28.20 (SD: 33.21)	9	6 (66.7%)	5 months		Loss to follow up (*n* = 30)

*AIC* Hemoglobin A1C, *FPG* Fasting Plasma Glucose test, *OGTT* Oral Glucose Tolerance Test, *FBG* Fasting Blood Glucose, *SD* Standard Deviation, *IQR* Interquartile range

The follow-up ranged from 3 to 6 months. The mean quality score using the NOS Scale was 7.1 (range 6-9). Two studies had a loss of follow-up larger than 10% [20, 27] and in one study it was unclear whether there was any loss to follow-up [19]. (Table 1: Description of the studies included in the systematic review and meta- analysis) and (Table 2: Newcastle Ottawa Score Assessment).

**Table 2:** Newcastle Ottawa Score Assessment

Cohort studies	Aftab et al (2017) [22]	Kishore et al (1971)	Gupta et al (2018) [21]	Oluboyo et al (1990) [18]	Basoglu et al(1999) [20]	Singh et al (1984) [17]	Boillat et al (2016)	Yan Lin et al (2017) [23]	Kornfeld et al, (2016) [19]	Kubjane et al (South Africa, 2019)	Philips et al (South Africa 2017) [24]
**Selection (maximum 4 points)**											
Representativeness of the exposed cohort	*		*	*	*	*		*	*	*	*
Selection of the non-exposed cohort?										*	
Ascertainment of exposure	*		*	*	*	*		*	*	*	*
Demonstration that outcome of interest was not present at start of study	*		*	*	*	*		*	*	*	*
**Comparability (maximum 2 points)**											
Comparability of cohorts on the basis of the design or analysis				*	*			*	*	*	*
Study controls for at least 3 additional risk factors?								*	*	*	
**Exposure (maximum 3)**											
Assessment of outcome/Assessment of outcome Independent blind assessment, record linkage	*		*	*	*	*		*	*	*	*
Was follow-up long enough for outcomes to occur?	*		*	*	*	*		*	*	*	*
Loss to follow up of NDM cohort < 10% or unclear	*		*	*		*		*		*	*
**Total score: 9**	6		6	7	6	6		8	7	9	7
**Case control studies**											
**Selection of cases and controls (maximum 4 points)**											
Case definition adequate		*					*				
Representativeness of the cases		*					*				
Selection of controls		*					*				
Definition of control		*					*				
**Comparability (maximum 2)**											
Comparability of cases and controls on the basis of the design or analysis		*					*				
Study controls for at least 3 additional risk factors											
**Exposure (maximum 3)**											
Ascertainment of exposure		*					*				
Same method cases and controls		*					*				
Non-response rate < 10% or unclear		*					*				
**Total (maximum 8)**		8					8				

* = yes

### Main findings of studies

Eleven studies identified reported on the reduction of TB-associated hyperglycemia over time and in all of these studies, it was possible to calculate the burden of unresolved hyperglycemia.

**Fig. 2:**
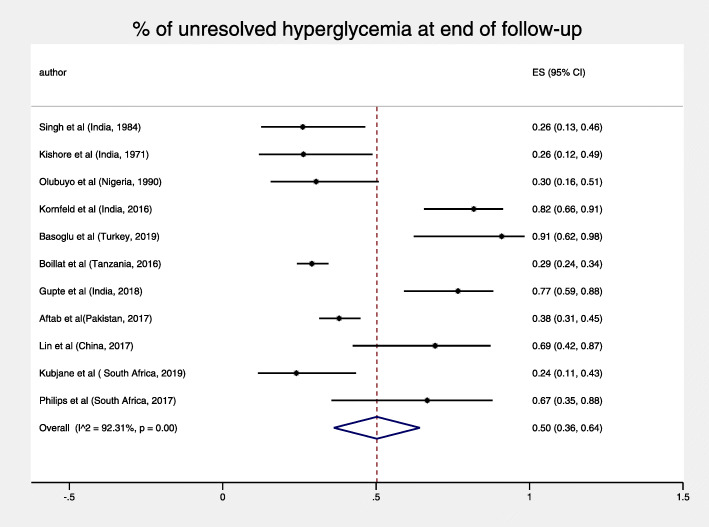
% of unresolved hyperglycemia at end of follow-up

Among the total study population of 2418 patients, (27,3) % had newly detected hyperglycemia at baseline. The pooled percentage of unresolved, newly detected cases of hyperglycemia at the end of follow up was 50% (95% CI: 36–64%) displayed a high heterogeneity I^2^ = 92.3%, *p* < 0.001 (Fig. 2).

**Fig. 3:**
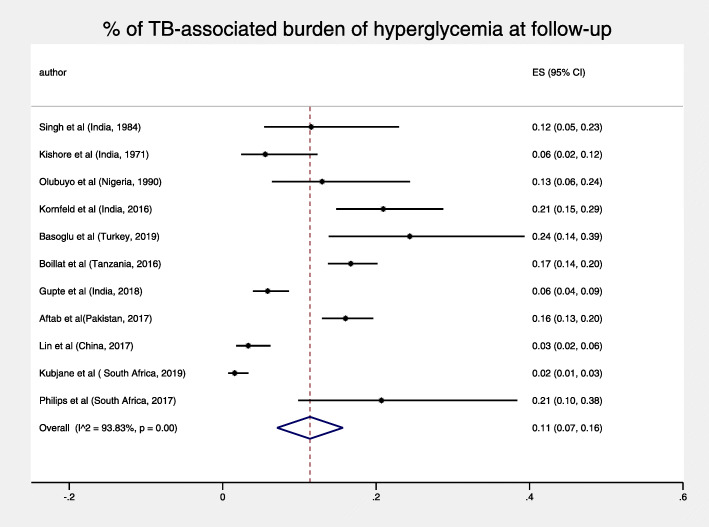
% of TB-associated burden of hyperglycemia at follow up

**Fig. 4:**
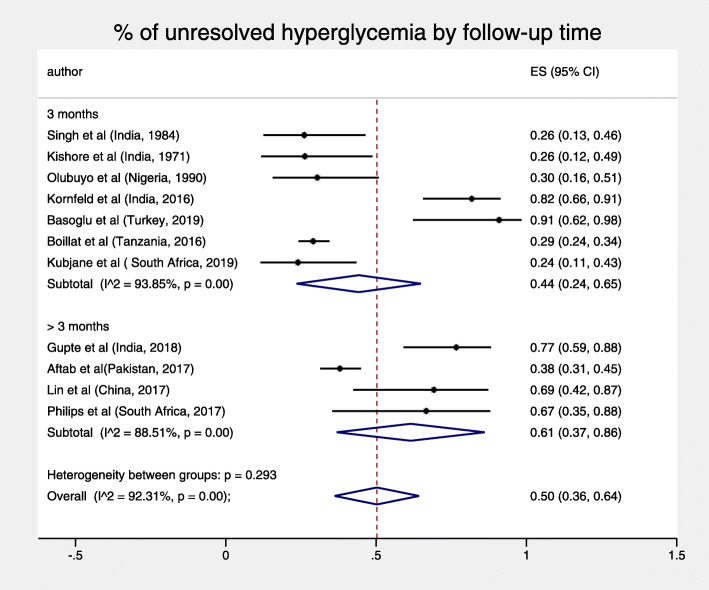
% of unresolved hyperglycemia by follow-up time

**Fig. 5:**
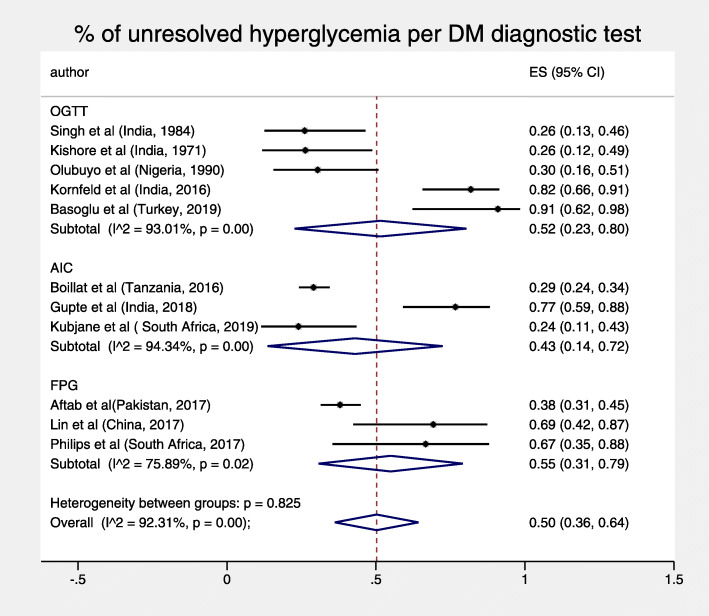
% of unresolved hyperglycemia per DM diagnostic test

**Fig. 6:**
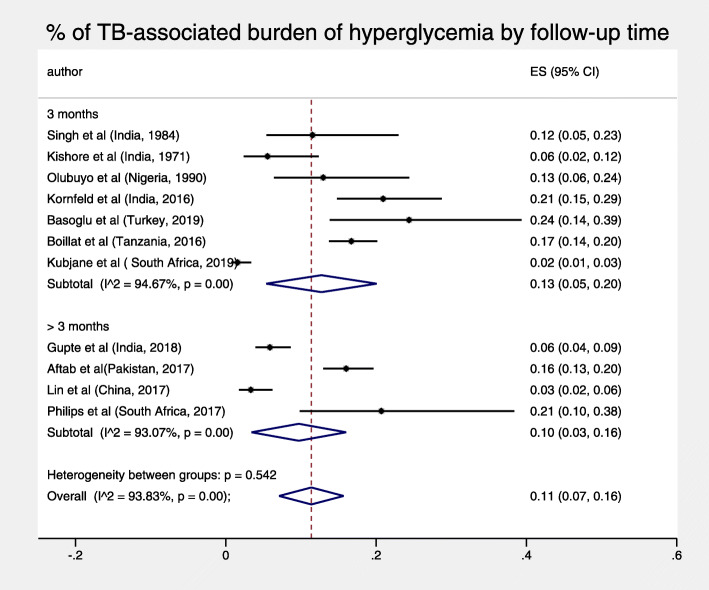
% of TB-associated burden of hyperglycemia by follow-up time

**Fig. 7:**
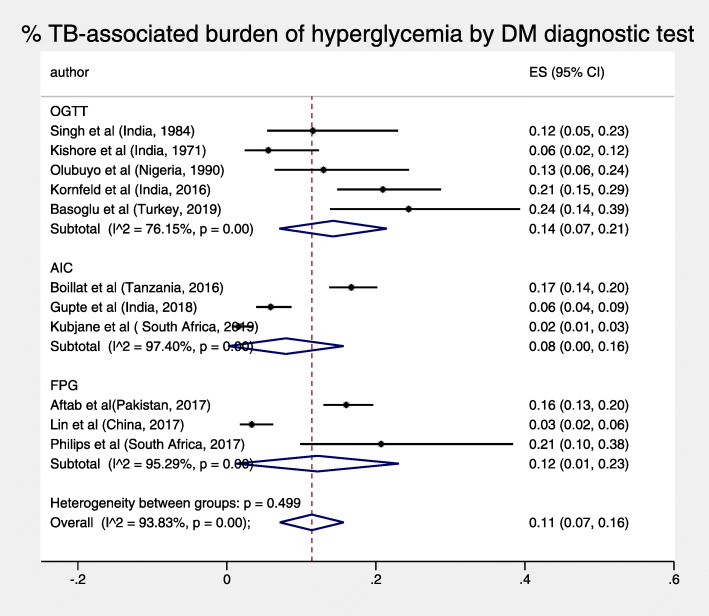
% of TB-associated burden of hyperglycemia by DM diagnostic test

The total pooled proportion of hyperglycemia in TB patients at 3 months or more was 11% (95% CI: 7–16%) and displayed high heterogeneity, I^2^ = 93.83% *p* < 0.001 (Fig. 3). For both objectives, sub-analyses by diagnostic test and duration of follow-up did not reduce heterogeneity, nor were both pooled figures substantially sensitive to the exclusion of any single study test (Figs. 4, 5, 6 and 7).

Only 2 studies identified risk factors for TB-associated hyperglycemia. Yan Lin et al. [18] reported an association between baseline characteristics such as HIV-positivity and smoking and unstable blood glucose levels during anti-TB treatment in a multivariate analysis, (aOR: 6.67, 95% CI: 1.24–35.71) and (aOR: 2.66, 95% CI: 1.37–5.2) respectively.

Using a different endpoint, Kubjane et al. [23] reported that HIV+ individuals were at higher odds of having newly diagnosed T2DM at baseline as diagnosed by AIC (aOR:1.7; 95%CI: 1.0–2.9), although at after 3- month of follow-up in both HIV-positive and HIV-negative participants, this association becomes statistically insignificant (aOR: 1.3, 95% CI: 0.2–8.1) versus (aOR: 2.4, 95% CI: 0.1–49.1) respectively [25].

## Discussion

To our knowledge, this is the first meta-analysis which both quantifies the burden of unresolved hyperglycemia in newly detected cases of hyperglycemia in TB patients over time and determines the total burden of hyperglycemia at follow- up. Although our meta-analysis demonstrated that the total burden of TB-associated hyperglycemia after a follow-up of 3 to 6 months is slightly above 10%, in regard to the total population who has embarked on TB treatment, hyperglycemia remains unresolved after follow-up in half of the patients with newly observed hyperglycemia at baseline.

It is noteworthy that our pooled figures be appreciated in light of the uncertainty of the length of follow-up required for TB-associated hyperglycemia to resolve by itself. Another element to consider is that studies did not estimate the proportion of the individuals presenting with newly detected hyperglycemia at the time of TB diagnosis who may have had undiagnosed T2DM or pre- T2DM prior to their TB diagnosis. However, one eligible study [20] reported that the 6.8% in the newly diagnosed T2DM group that did not change much with TB treatment were very likely patients with longstanding, undiagnosed T2DM.

A meta-analysis could not be performed for the third objective, i.e. quantifying risk factors for unresolved hyperglycemia, as there were only 2 studies identified, which explored risk factors for persistent hyperglycemia in newly observed cases. HIV was found to be an independent risk factor for unstable FGB [18], and for having newly detected hyperglycemia at baseline but not at follow-up [25] however, as HAART has been linked to hyperglycemia in HIV-infected patients, it is unsure whether this is due to HAART or TB or anti TB drugs [25, 28]. It is also noteworthy, that the sample size of HIV infected patients was low (*n* = 9). Also, smoking was found to be an independent risk factor for unstable FGB [18], which is consistent with findings of a meta-analysis that active smokers had an increased risk of T2DM compared with nonsmokers, with a pooled RR of 1.38, (95% CI: 1.28–1.49) [29].

Also, at baseline 2 studies [17, 22] in our review suggested an association between extensive pulmonary TB disease/higher smear grade and newly screened hyperglycemia and two studies suggested a higher median age of patients with newly screened hyperglycemia than those with euglycemia [20, 21].

As there were few studies identified with a follow-up period between 3 and 6 months, aligned with TB management, it remains to be elucidated why there were no differences in burden between a follow-up of 3 months and three to 6 months. One hypothesis is that it may be due to a lack of benefits of extending follow up of new cases of hyperglycemia beyond 3 months or another hypothesis may be that hyperglycemia requires a longer follow-up than 6 months to resolve. Future studies should aim to explore the optimum time of follow-up for patients with TB and hyperglycemia.

Our sub-analysis did not reveal any statistically significant differences between different periods of follow up and different DM tests, suggesting that heterogeneity may not have significantly been caused by these factors. The high heterogeneity may be attributed to the varied prevalence of TB comorbidities, including smoking, undernutrition, and vitamin D deficiency which are also associated with diabetes [30–32], hence may have affected TB severity and resulted in unresolved hyperglycemia. Only 4 studies described patient demographic, lifestyle, and anthropometric details at enrollment by glycemic status, including newly detected hyperglycemic patients [18, 20, 21, 26].

### Strengths and limitations

To our knowledge, this is the first systematic review which attempts to quantify unresolved hyperglycemia in patients where it is newly detected, through a search of several databases. A strength of this study is that it includes studies from different settings and median age, without any restriction on time period, which in turn enabled us to capture a more representative population. Another strength is that, by including only prospective studies in the analysis, the temporal criterion of causality is fulfilled. Furthermore, the mean NOS quality score assigned was high.

The results should be interpreted in light of a number of limitations, including the small number of studies and small sample size of single studies which results in large confidence intervals, which in turn precludes us from making appropriate inferences. Although we were interested in the percentage of change of newly detected hyperglycemia, the strengths and limitations of the different tests should be taken into account. Since use of FBG alone to diagnose hyperglycemia can underestimate the prevalence of impaired glucose tolerance by as much as 50% compared with the gold standard test i.e. the cumbersome but more accurate oral glucose tolerance test [33], the actual prevalence of hyperglycemia among TB patients in all studies using FBG as diagnostic parameter may have been underestimated. In addition, since the diagnostic parameter HbA1C measures the average blood glucose levels over a three-month span, it may not be able to reflect the glycemic fluctuations during the follow up period [10], although Kornfeld et al. (2016) [20] found statistically significant and clinically meaningful changes in HbA1c between baseline and at month 3. It is noteworthy, that albeit the NOS quality score was high, as the required follow up for unresolved TB-associated hyperglycemia remains to be determined, the quality of the studies may be slightly overestimated. Another limitation may be the high heterogeneity between studies, which can be considered as a tradeoff for having a heterogeneous population. Furthermore, despite our attempts to contact medical institutions in India, we have not been able to access neither the abstracts, nor the full text of 6 studies published in Indian journals.

### Public health and clinical implications

Our pooled effect size suggests that if T2DM is suspected during the acute phase of TB, this may result in over-diagnosis and consequently over-treatment in about 50% of patient, in whom abnormal glucose tolerance is reversible. However, the non-negligible proportion of unresolved cases of hyperglycemia at the end of follow up underscores the necessity to repeat DM screening later within the course of TB treatment in those with newly detected TB-associated hyperglycemia.

There is also some evidence suggesting that patients with more extensive pulmonary TB disease, may have increased TB-associated stress, which in light of the growing number of MDR TB across the world, may have vast implications.

Our review has enabled us to identify some epidemiological and operational research gaps.

### Epidemiological research gaps

#### Increased risk of T2DM

Studies are needed to assess whether TB patients with transient hyperglycemia are at increased risk of developing T2DM later [34], such as women with a history of Gestational Diabetes (GDM), who have shown to have at least 7-fold higher risk of T2DM later in life compared to women without GDM [35]. Moreover, in order to assess the risk of progression from persistent hyperglycemia to overt T2DM, there may be a need for studies with a longer follow-up time than 6 months.

#### Screening protocol

More research with multiple measurements during the course of TB treatment and after 6 months is required to define the optimal timing of monitoring for T2DM.

#### Impact of HIV

As both HIV infection and antiretroviral drugs have metabolic effects, more studies should focus on the longitudinal course of glycemia during tuberculosis treatment in high HIV prevalence settings.

#### Multi drug resistant TB (MDR-TB)

Our review suggests that increased TB-associated stress may accentuate hyperglycemia. In light of a growing epidemic of MDR-TB, the impact of MDR TB-associated stress on the resolution of hyperglycemia must also be investigated.

#### Mathematical modelling

A data-driven modelling approach may help understand the impact of the TB on T2DM and determine the optimal management of the two diseases, given that management of one disease has an impact on the other. Furthermore, it might be possible to develop a statistical model to understand the significant correlation or association of risk factors (HIV positivity, smoking, and MDR-TB) with unresolved TB associated hyperglycemia.

#### Operational research

Another issue in bi-directional screening is the need for decentralized T2DM programs that would allow T2DM patients to be tracked post diagnosis [36]. Given the escalating proportions of TB and T2DM comorbidity in Asian countries and in HIV prevalence settings, more evidence is urgently needed on how to implement TB associated hyperglycemia monitoring within these settings.

## Conclusion

Our review suggested that although in more than half of all patients the newly detected hyperglycemia when initiating TB treatment is unresolved, the total burden of patients who exhibited unresolved hyperglycemia after a follow-up period of 3 to 6 months is small. This finding underscores the need to continue monitoring alongside the TB treatment to early identify patients who do not compensate their level of blood glucose in order to avoid long terms complications.

Early identification of individuals at risk for unresolved hyperglycemia would reduce DM disease complications and thereby help determine who would benefit from glucose-lowering interventions. Studies are warranted to assess whether TB patients with unresolved hyperglycemia having certain baseline characteristics such as HIV positivity, smoking, and MDR-TB require longer follow up and are at heightened risk of developing T2DM at a later stage in life.

## Supplementary information

**Additional file 1.** Search strategy.

**Additional file 2.** PRISMA 2009 Checklist.

## Data Availability

All data generated or analyzed during this study are included in the supplementary information files.

## Abbreviations

DM: Diabetes Mellitus
FBG: Fasting Blood Glucose
GDM: Gestational Diabetes Mellitus
HIV: Human Immunodeficiency Virus
MDR-TB: Multi drug resistant Tuberculosis
TB: Tuberculosis
WHO: World Health Organization
