# Supplementary material for: The epidemiology of tuberculosis-associated hyperglycemia in individuals newly screened for type 2 diabetes mellitus: systematic review and meta-analysis
Source: BMC Infect Dis. 2020 Dec 9;20:937. doi: 10.1186/s12879-020-05512-7 (PMC7724718; doi:10.1186/s12879-020-05512-7)
Supplement: Supplementary file 1 — Additional file 1. Search strategy. [file 12879_2020_5512_MOESM1_ESM.docx]

Requester: Mbabazi Kariisa

Aug 17 20

Searched Performed by: Yolanda Strayhorn

We are interested in hyperglycemia/or DM caused by TB.

Search Topic:

Tuberculosis"[Mesh] OR "tuberculosis" AND "Hyperglycemia"[MESH] AND "diabetes" ; ( "tuberculosis" AND ( hyperglycemia OR diabetes ) ) AND ( ( AND ( tuberculosis ) ) OR ( diabetes AND mellitus ) AND ( LIMIT-TO ( DOCTYPE , "ar" ) ) AND ( LIMIT-TO ( LANGUAGE , "English" ) )

Run the updated search for the full time period (1960 – Dec 11, 2019) that would be great.

Document type – AR – Scopus

Mbabazi Kariisa 121919

| **Database** | **Strategy** | **Run Date** | **Records** |
| --- | --- | --- | --- |
| **Embase**  **(OVID)**  **1947-** | Tuberculosis/ or ((tubercul* or tuberculosis or tuberculous).ab,ti.  AND  hyperglycaemia/ OR Diabetes Mellitus/ or (hyperglycaemia OR hyperglycemia or diabetic* or diabetes or (diabetes adj2 mellitus) or (metabolic adj2 syndrome) or (insulin adj2 resistan*)).ab,ti.  AND  limit to (english language and yr="1960 -Current") | 12/19/19 | **1002**  **-21**  **duplicates**  **= 981**  **unique items** |
| **Global Health**  **(OVID)**  **1947-** | Tuberculosis/ or ((tubercul* or tuberculosis or tuberculous or tuberculosis) adj2 induced).ab,ti.  AND/OR  hyperglycaemia/ or Diabetes Mellitus/ or (hyperglycemia or diabetic* or diabetes or (diabetes adj2 mellitus) or (metabolic adj2 syndrome) or (insulin adj2 resistan*)).ab,ti.  limit 5 to (english language and yr="1960 -Current") | 12/19/19 | **1516**  **-204**  **duplicates**  **= 1312**  **unique items** |
| **PubMed** | ((("hyperglycemia"[MeSH Terms] OR "diabetes mellitus"[MeSH Terms]) OR ((((hyperglycemia[Title/Abstract] OR diabetic[Title/Abstract] OR diabetics[Title/Abstract] OR diabetes[Title/Abstract]) OR diabetes mellitus[Title/Abstract]) OR metabolic syndrome[Title/Abstract]) OR insulin resistance[Title/Abstract])) AND ("tuberculosis"[MeSH Terms] OR (tuberculosis[Title/Abstract] OR tuberculous[Title/Abstract]))  AND  ("1960/01/01"[PDAT] : "2019/12/31"[PDAT])) AND "english"[Language] | 12/20/19 | **2736**  **-1188**  **duplicates**  **= 1548**  **unique items** |
| **Scopus**  1966 - present | (INDEXTERMS("Tuberculosis") OR TITLE-ABS("tuberculosis") OR TITLE-ABS("tuberculous")  AND  (INDEXTERMS("Hyperglycemia") OR INDEXTERMS("Diabetes Mellitus") OR (TITLE-ABS("hyperglycemia") OR TITLE-ABS("diabetic*") OR TITLE-ABS("diabetes") OR (TITLE-ABS("diabetes") W/2 TITLE-ABS("mellitus")) OR (TITLE-ABS("metabolic") W/2 TITLE-ABS("syndrome")) OR (TITLE-ABS("insulin") W/2 TITLE-ABS("resistan*"))))  ( LIMIT-TO ( LANGUAGE , "English" ) )  Dates 01/01/1960 or 12/31/20 | 12/20/19 | **5890**  **-3175**  **duplicates**  **= 2715**  **unique items** |

 Notes: Duplicates were identified using the Endnote automated "find duplicates" function with preference set to match on title, author and year, and removed from your Endnote library. There will likely be additional duplicates found that Endnote was unable to detect.
